# The nonlinear association between the triglyceride glucose-waist-to-height ratio index and cardiovascular disease among middle-aged and older adults in China with cardiovascular-kidney-metabolic syndrome stages 0–3

**DOI:** 10.3389/fcvm.2025.1604270

**Published:** 2025-08-29

**Authors:** Cheng Huang, Jing Jia, Yuanyuan He

**Affiliations:** ^1^ Department of Colorectal Surgery, First People’s Hospital of Xiaoshan District, Hangzhou, Zhejiang, China; ^2^ Department of Emergency, First People’s Hospital of Xiaoshan District, Hangzhou, Zhejiang, China; ^3^ Department of Anesthesiology, First People’s Hospital of Xiaoshan District, Hangzhou, Zhejiang, China

**Keywords:** TyG-WHtR, cardiovascular disease, Cardiovascular-Kidney-Metabolic syndrome, Chinese, CHARLS

## Abstract

**Background:**

Cardiovascular disease (CVD) poses a critical challenge to global public health, especially in individuals with Cardiovascular-Kidney-Metabolic (CKM) syndrome. The association between the triglyceride glucose-waist-to-height ratio index (TyG-WHtR) and CVD among middle-aged and older Chinese adults with CKM stages 0–3 is not well understood.

**Methods:**

Data were obtained from the China Health and Retirement Longitudinal Study (CHARLS), a nationally representative longitudinal study of individuals aged 45 years and older. Cox proportional hazards models were constructed to evaluate the association between TyG-WHtR and CVD risk. Subgroup analyses were conducted among different groups to evaluate consistency of findings and explore potential interactions. Restricted cubic spline regression was employed to evaluate the dose-response relationship.

**Results:**

During a mean follow-up period of 7.77 ± 2.13 years, 1,595 participants (23.58%) developed CVD. After adjusting for covariates, TyG-WHtR was significantly associated with CVD risk (HR: 1.09, 95% CI: 1.01–1.18). Notably, this relationship was independent of other variables and displayed a nonlinear pattern, with an inflection point identified at 3.57. Beyond this threshold, the adjusted HR increased to 1.17 (95%CI: 1.08–1.71).

**Conclusion:**

TyG-WHtR demonstrates a nonlinear relationship with CVD among middle-aged and older Chinese adults with CKM stages 0–3.

## 1 Introduction

Cardiovascular disease (CVD) is the foremost contributor to global mortality, accounting for 32% of fatalities in 2019 (1). Although the global CVD mortality rate declined by 14.5% from 2000 to 2019, the absolute burden of the disease continues to rise, particularly in Asia (2). In China, CVD-related disability-adjusted life years increased by 58.5% between 1990 and 2019, highlighting the urgent need for improved risk assessment and prevention strategies tailored to the Chinese population (3).

The pathophysiological relationship between metabolic disorders and CVD has been extensively investigated, with obesity and insulin resistance emerging as critical modifiable risk factors. Body mass index (BMI) and waist circumference (WC) have shown limited effectiveness in accurately representing metabolic risks associated with central adiposity (4). The waist-to-height ratio (WHtR) demonstrates a more robust correlation with CVD (5). The WHtR is more effective than alternative body measurement methods in predicting cardiovascular outcomes, significantly exceeding both BMI and WC (6). At the same time, triglyceride-glucose (TyG) has emerged as a trustworthy indicator of insulin resistance (7, 8). Numerous studies across diverse Asian populations, including those from China, Thailand, and South Korea, have consistently demonstrated an independent association between TyG and CVD (9–11). These results highlight the necessity of incorporating both lipid metabolism and glycemic control in the evaluation of cardiovascular risk.

Recently, a composite marker that combines TyG and WHtR, known as TyG-WHtR, has been introduced to encapsulate glucose and lipid metabolism disorders along with central obesity into a unified measurement. A study from the US, UK, and China indicates that among hypertensive populations, TyG and its related indices are associated with CVD in a U-shaped pattern (12). In populations with metabolic abnormalities, the advantages of TyG-WHtR remain evident (13, 14). In 2013, the American Heart Association first conceptualized Cardiovascular-Kidney-Metabolic (CKM) syndrome, integrating the complex interconnections among metabolic risk factors, chronic kidney disease (CKD), and cardiovascular disease (15, 16). A 2024 study established a linear relationship between TyG-BMI and CVD specifically within the CKM stages 0–3 population (17). Therefore, we hypothesized that TyG-WHtR exhibits a dose-response relationship with CVD incidence in this cohort and aimed to identify potential inflection points.

In this study, we utilized a substantial longitudinal cohort derived from the China Health and Retirement Longitudinal Study (CHARLS) to analyze the dose-response correlation between TyG-WHtR and CVD among middle-aged and older Chinese adults with CKM stages 0–3.

## 2 Methods

### 2.1 Study design and population

CHARLS is a prominent nationally representative longitudinal survey targeting individuals aged 45 years and older. The initial assessment (2011–2012) employed stratified sampling methodology spanning 28 provinces, encompassing 150 counties and 450 communities across mainland China. Additional surveys were completed in 2013, 2015, 2018, and 2020 to document evolving health, social, and economic metrics. This research received approval from Peking University's Biomedical Ethics Committee (IRB00001052-11015) and secured informed consent from participants.

**Figure 1:**
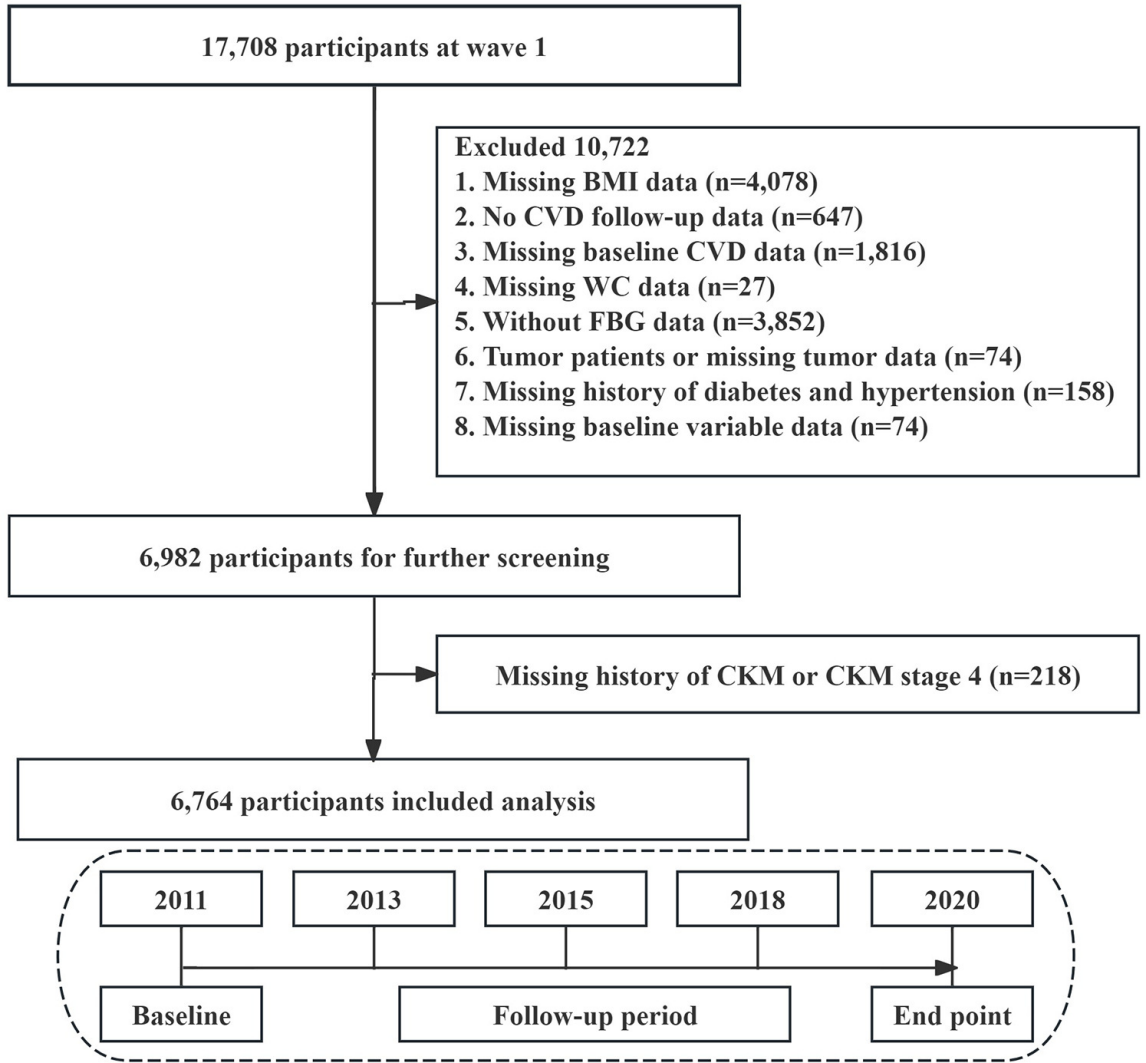
Flowchart of the selection process of study subjects.

We used data from the first wave (2011–2012) for this analysis, focusing on participants for whom blood sample data were available. The data analysis excluded cases under the following criteria: those lacking BMI data (*n* = 4,078), participants without CVD follow-up information (*n* = 647), participants missing baseline CVD details (*n* = 1,816), those without WC data (*n* = 27), participants lacking FBG data (*n* = 3,852), tumor patients or participants with missing tumor information (*n* = 74), participants lacking a history of diabetes and hypertension data (*n* = 158), and instances missing baseline variable data (*n* = 74). Finally, participants without CKM data and those in CKM stage 4 were excluded (*n* = 218). 6,764 participants were included in this study for analysis (Figure 1).

### 2.2 Determination of CVD and calculation of TyG-WHtR

In this study, CVD constituted the principal outcome of interest. CVD status was determined based on participants' self-reported medical histories, elicited through a structured health questionnaire during face-to-face interviews. Study subjects reported whether a healthcare professional had diagnosed them with heart failure, myocardial infarction, angina, coronary heart disease, or stroke (18). A positive response to any of these conditions led to the participant's classification as having CVD.

TyG-WHtR = Ln[TG (mg/dl) × glucose (mg/dl)/2] × [WC (cm)/height (cm)] (19).

### 2.3 Definition of CKM syndrome stages 0 to 4

The staging framework for Cardiovascular-Kidney-Metabolic (CKM) syndrome classifies disease progression across five distinct phases (0–4):

Stage 0: Absence of recognized CKM risk factors;
Stage 1: Characterized by excess adiposity (abdominal obesity) and/or impaired glucose regulation (prediabetes);
Stage 2: Manifestation of confirmed metabolic dysfunction (including type 2 diabetes, hypertension, hypertriglyceridemia) or renal impairment;
Stage 3: Presence of asymptomatic cardiovascular pathology within the CKM context
Stage 4: Established clinical cardiovascular disease (encompassing coronary artery disease, heart failure, cerebrovascular events, peripheral arterial disease, or atrial fibrillation) with underlying CKM pathophysiology (20).

### 2.4 Ascertainment of covariates

Our investigation utilized the CHARLS dataset, which provides extensive health-related variables for participants aged 45 and above. Professional interviewers conducted standardized data collection procedures. Comprehensive data collection encompassed multiple domains. Demographic characteristics included age, gender, educational attainment, marital status (married/unmarried), medication history (yes/no), and residential environment (rural/urban). Current lifestyle behaviors, particularly smoking status and alcohol consumption patterns, were systematically documented. Anthropometric measurements were conducted under standardized conditions, with participants instructed to remove shoes and heavy clothing before assessment of height, weight, and blood pressure parameters.

Blood specimen collection followed a mandatory minimum 8 h fasting period. Laboratory analyses employed standardized enzymatic methodologies to quantify multiple biochemical parameters: fasting blood glucose (FBG), high-density lipoprotein cholesterol (HDL-C), low-density lipoprotein cholesterol (LDL-C), total cholesterol (TC), triglycerides (TG), uric acid (UA), HbA1c, blood urea nitrogen (BUN), and C-reactive protein (CRP). All research procedures strictly adhered to established ethical guidelines and protocols throughout the study duration. The covariates included in this study were carefully selected, building on the methodologies and analytical frameworks utilized in previously published research that also analyzed data from the CHARLS database (21). Additionally, cardiovascular-related indicators, such as UA, CRP, and BUN, were included in the analysis (22–24).

### 2.5 Statistical analysis

Summary statistics included means ± standard deviations or medians (interquartile ranges) for continuous variables, and frequencies (percentages) for categorical data. Between-group differences were assessed using one-way ANOVA or Kruskal–Wallis tests for continuous variables, and Chi-square tests for categorical variables. We evaluated multicollinearity through Variance Inflation Factor (VIF) calculations, excluding variables with VIF values exceeding 5 from subsequent analyses (Supplementary Table S1). Our statistical framework employed multiple approaches to examine metabolic parameters and diabetes relationships. The investigation began with univariate Cox regression, followed by comprehensive multivariate analysis. Three models were established: Model I (unadjusted), Model II (adjusted for demographic and clinical factors such as marital status, rural residence, hypertension, drinking status, smoking status, medication history, CKM, SBP, DBP, age, and education), and Model III (further adjusted for biochemical markers, including HDL-C, LDL-C, CRP, BUN, UA, and HbA1c). To evaluate result consistency, we conducted stratified analyses across various population subsets: age groups (<60 vs. ≥60 years), gender categories, BMI classifications (<24 kg/m^2^ vs. ≥24 kg/m^2^), smoking and alcohol consumption patterns, and presence of metabolic conditions including diabetes and dyslipidemia. Potential interactions between subgroup variables were also assessed. Dose-response relationships underwent examination through restricted cubic spline analysis with four knots, utilizing two-segment logistic regression to identify critical threshold points. Statistical analyses utilized EmpowerStats and R software, with significance determined by a two-tailed *p*-value threshold of 0.05.

## 3 Results

### 3.1 Baseline characteristics of participants

**Figure 2:**
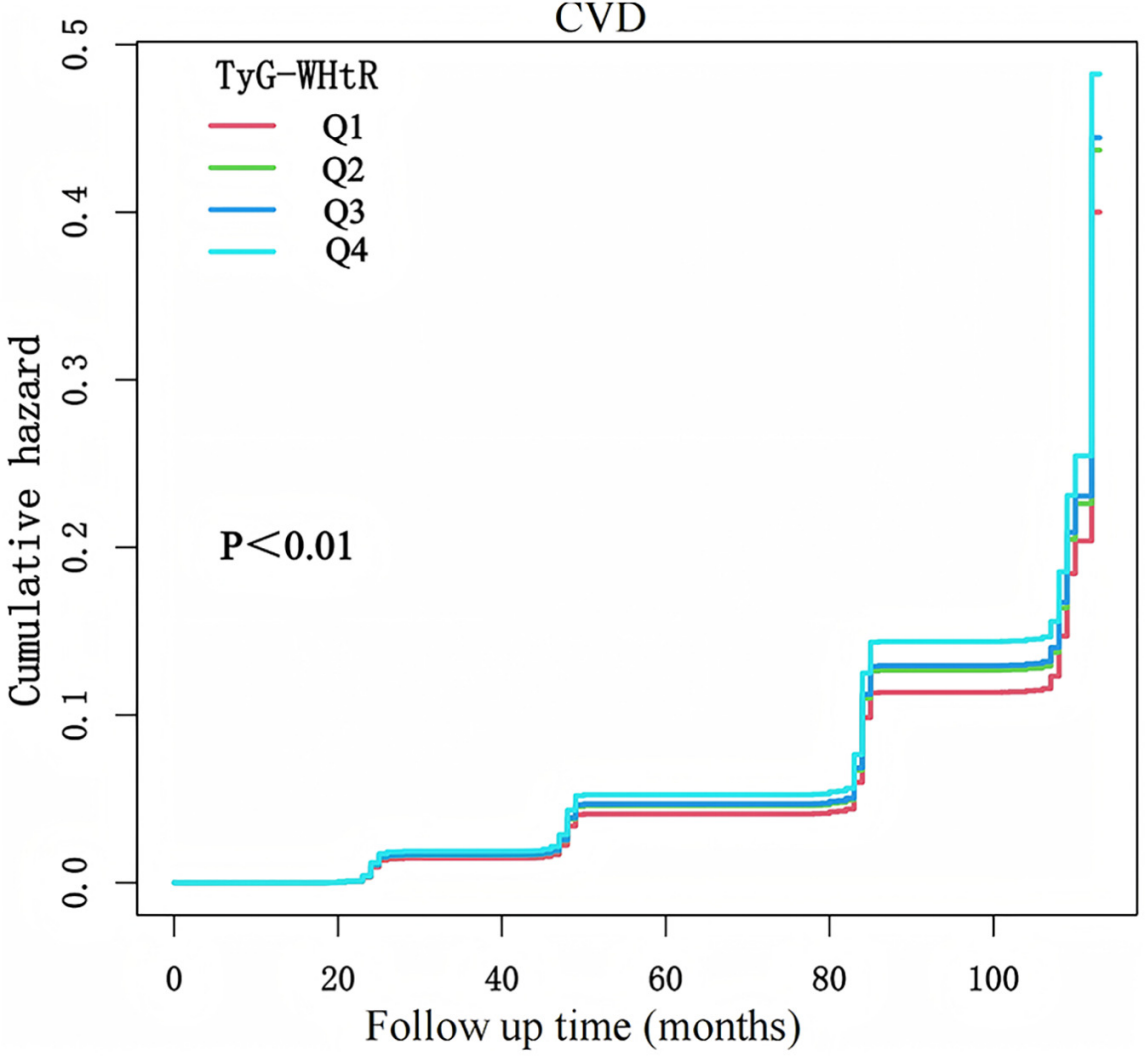
K-M plot of CVD incidence by TyG-WHtR.

This study included 6,764 participants, comprising 3,620 females and 3,144 males, with an average age of 58.47 ± 9.50 years. Over a follow-up period of 7.77 ± 2.13 years, 1,595 individuals (23.58%) developed CVD. Participants in the highest TyG-WHtR quartile (Q4) exhibited markedly worse metabolic parameters and higher CVD prevalence compared to lower quartiles (Table 1). Notably, cardiometabolic comorbidities increased progressively across quartiles, with hypertension prevalence rising from 30.51% (Q1) to 61.92% (Q4), diabetes from 5.38% to 28.86%, and dyslipidemia from 3.14% to 12.90%. An incremental trend was observed between TyG-WHtR levels and CVD risk (Figure 2). CVD prevalence nearly doubled from Q1 (18.10%) to Q4 (30.46%).

**Table 1:** Baseline characteristics of study population.

Characteristic	Q1 (<4.09)	Q2 (4.09 to 4.58)	Q3 (4.59 to 5.13)	Q4 (>5.13)	*P*-value
Subjects, n	1,691	1,691	1,691	1,691	
Age, years	58.35 ± 9.60	57.93 ± 9.50	58.27 ± 9.43	59.34 ± 9.44	<0.001
Gender					<0.001
Female	602 (35.60%)	831 (49.14%)	977 (57.78%)	1,210 (71.56%)	
Male	1,089 (64.40%)	860 (50.86%)	714 (42.22%)	481 (28.44%)	
Married, *n* (%)	1,504 (88.94%)	1,519 (89.83%)	1,514 (89.53%)	1,487 (87.94%)	0.304
Rural, *n* (%)	1,221 (72.21%)	1,172 (69.31%)	1,062 (62.80%)	992 (58.66%)	<0.001
SBP, mmHg	123.71 ± 19.88	126.69 ± 20.09	130.75 ± 20.52	136.90 ± 21.70	<0.001
DBP, mmHg	72.28 ± 11.59	74.09 ± 11.82	76.28 ± 11.86	79.01 ± 11.80	<0.001
BUN, mg/dl	16.24 ± 4.62	15.61 ± 4.50	15.61 ± 4.52	15.27 ± 4.26	<0.001
FBG, mg/dl	99.22 ± 17.96	103.12 ± 22.54	107.93 ± 29.03	124.05 ± 47.15	<0.001
TC, mg/dl	182.75 ± 35.79	189.39 ± 34.90	197.26 ± 36.50	205.31 ± 40.22	<0.001
TG, mg/dl	78.28 ± 39.60	100.58 ± 47.10	125.76 ± 59.03	202.23 ± 134.06	<0.001
HDL-C, mg/dl	59.49 ± 15.60	54.51 ± 14.61	50.01 ± 13.28	43.14 ± 11.93	<0.001
LDL-C, mg/dl	109.62 ± 31.57	116.21 ± 31.33	123.06 ± 33.30	119.22 ± 40.39	<0.001
CRP, mg/dl	2.35 ± 7.52	2.75 ± 9.42	2.49 ± 6.89	2.78 ± 4.63	0.261
HbA1c %	5.08 ± 0.50	5.15 ± 0.59	5.25 ± 0.71	5.57 ± 1.12	<0.001
UA, mg/dl	4.29 ± 1.16	4.32 ± 1.19	4.41 ± 1.28	4.61 ± 1.27	<0.001
Hypertension, *n* (%)	516 (30.51%)	628 (37.14%)	816 (48.26%)	1,047 (61.92%)	<0.001
Diabetes, *n* (%)	91 (5.38%)	144 (8.52%)	239 (14.13%)	488 (28.86%)	<0.001
Dyslipidemia, *n* (%)	51 (3.02%)	74 (4.38%)	127 (7.51%)	242 (14.31%)	<0.001
Drinking, *n* (%)	814 (48.14%)	692 (40.92%)	650 (38.44%)	511 (30.22%)	<0.001
Smoking, *n* (%)	911 (53.87%)	695 (41.10%)	582 (34.42%)	417 (24.66%)	<0.001
Education					0.158
Lower level	1,169 (69.13%)	1,169 (69.13%)	1,167 (69.01%)	1,218 (72.03%)	
Higher level	522 (30.87%)	522 (30.87%)	524 (30.99%)	473 (27.97%)	
Medication history					<0.001
No	1,217 (71.97%)	1,177 (69.60%)	1,107 (65.46%)	931 (55.06%)	
Yes	474 (28.03%)	514 (30.40%)	584 (34.54%)	760 (44.94%)	
CKM					<0.001
0	216 (12.77%)	75 (4.44%)	3 (0.18%)	0 (0.00%)	
1	173 (10.23%)	294 (17.39%)	252 (14.90%)	64 (3.78%)	
2	220 (13.01%)	408 (24.13%)	565 (33.41%)	682 (40.33%)	
3	1,082 (63.99%)	914 (54.05%)	871 (51.51%)	945 (55.88%)	

BUN, blood urea nitrogen; FBG, fasting blood glucose; TC, total cholesterol; HDL-C, high-density lipoprotein cholesterol; LDL-C, low-density lipoprotein cholesterol; CRP, C-reactive protein; UA, uric acid.

### 3.2 Association between TyG-WHtR and CVD

**Figure 3:**
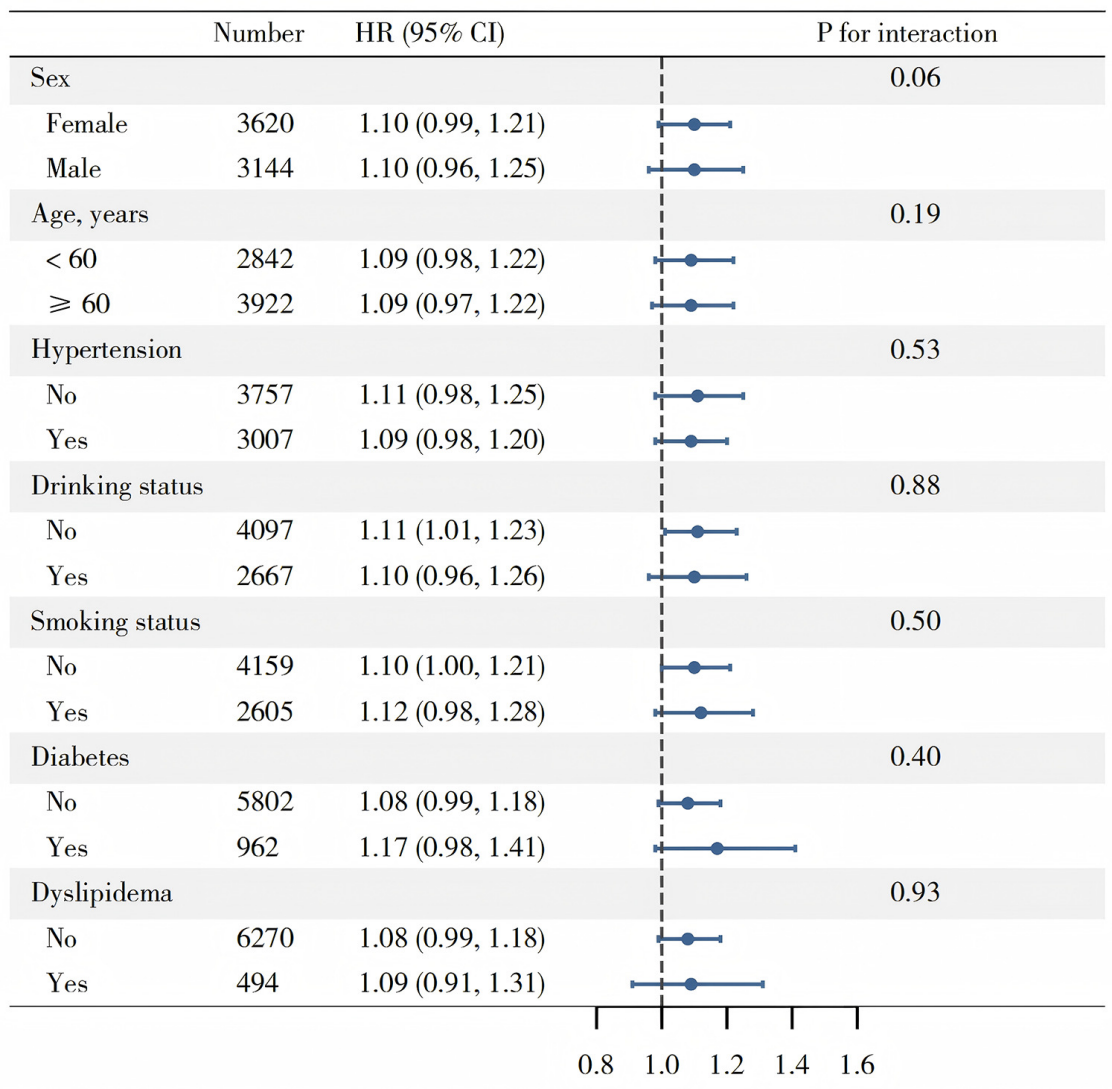
Subgroup analysis of associations between TyG-WHtR and CVD.

Multivariate Cox regression analysis was employed to evaluate the association between TyG-WHtR and CVD (Table 2). Following the multicollinearity analysis, all variables exhibited VIF below 5. In Model 1, without adjusting for covariates, each one standard deviation increase in TyG-WHtR was associated with a 29% increased risk of CVD (HR: 1.29, 95% CI: 1.22–1.37). After sequential adjustment for sociodemographic factors, blood pressure, lipid profiles, and inflammation-related indicators in Models 2 and 3, the association remained significant, with the HR decreasing slightly to 1.10 (95% CI: 1.03–1.17) in Model 2 and 1.09 (95% CI: 1.01–1.18) in Model 3. A trend test within the multivariate Cox regression analysis indicated a statistically significant relationship. Moreover, within CKM stages 0–4, we also arrived at similar conclusions (Supplementary Table S2). To prevent reverse causality, we excluded participants whose follow-up time was under 2 years. The findings from this subgroup were consistent with the main results (Supplementary Table S3). Subgroup analysis showed no significant interactions that modified the association between TyG-WHtR and CVD (Figure 3).

**Table 2:** Multivariate Cox regression analyses for the association between TyG-WHtR and CVD across CKM stages 0–3.

		HR (95% CI)	
	Model 1	Model 2	Model 3
TyG-WHtR	1.29 (1.22, 1.37)	1.10 (1.03, 1.17)	1.09 (1.01, 1.18)
TyG-WHtR quartile			
Q1	Ref	Ref	Ref
Q2	1.19 (1.02, 1.38)	1.14 (0.97, 1.33)	1.12 (0.96, 1.31)
Q3	1.37 (1.18, 1.59)	1.17 (1.00, 1.37)	1.15 (0.98, 1.35)
Q4	1.80 (1.56, 2.08)	1.31 (1.11, 1.54)	1.28 (1.07, 1.54)
*P*-trend	<0.001	<0.001	<0.001

Model 1: No covariates were adjusted. Model 2: Age, sex, marital status, rural, hypertension, dyslipidemia, diabetes, drinking status, smoking status, CKM, medication history, SBP, DBP were adjusted. Model 3: HDL-C, LDL-C, TC, UA, BUN, HbA1c and CRP were further adjusted based on model 2.

### 3.3 Nonlinear association between TyG-WHtR and CVD

**Figure 4:**
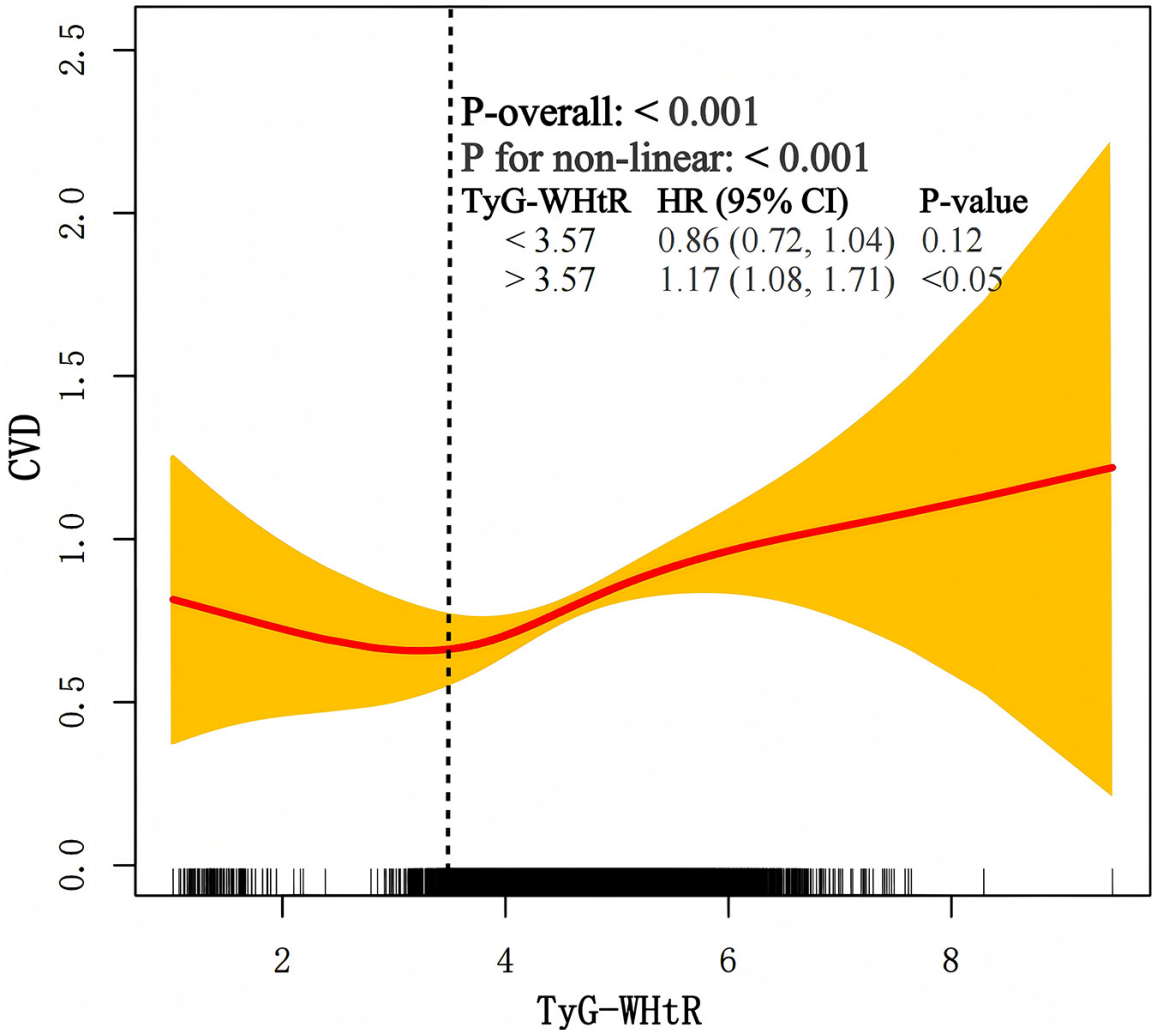
The nonlinear association between TyG-WHtR and CVD.

A restricted cubic spline demonstrated a nonlinear association between TyG-WHtR and CVD (Figure 4). The inflection point, calculated using model 3, was determined to be 3.57. When TyG-WHtR was below the threshold of 3.57, HR was 0.90 (95% CI: 0.72–1.04). When TyG-WHtR exceeded 3.57, each standard deviation increase was associated with a 17% increase in the risk of CVD (HR: 1.17, 95% CI: 1.08–1.71).

## 4 Discussion

This extensive cohort investigation, utilizing long-term follow-up data from CHARLS, uncovered three key findings. First, a significant association was established between TyG-WHtR and CVD. Secondly, the association between TyG-WHtR and CVD demonstrated a nonlinear pattern. Third, the RCS analysis indicated an inflection point at 3.57.

In 2016, Sánchez-Íñigo et al. (25) first introduced the concept of the TyG index, demonstrated a positive association between TyG and CVD. Given the strong connection between obesity and CVD, subsequent studies have increasingly examined TyG alongside obesity-related indicators to assess its association with CVD events. Currently, markers like TyG-BMI, TyG-WC, and TyG-WHtR are increasingly recognized as potential alternative indicators of insulin resistance (IR) (26, 27). Additionally, WHtR has been established as an effective measure for assessing central obesity and has shown a linear association with ischemic cardiovascular disease and ischemic stroke (28). A study conducted in the United States further confirmed that TyG-WHtR demonstrated a significant association with CVD-related mortality in NAFLD patients, with a HR of 2.22 (95% CI: 1.42–3.47) (14).

Our study also demonstrates a positive association between TyG-WHtR and CVD, aligning with results reported in earlier research (29). Earlier research using the CHARLS database also explored this relationship; however, it was limited to a cross-sectional design due to the absence of longitudinal data (21). A cohort study utilizing 12.5 years of UK Biobank data has shown that in patients with metabolic abnormalities, TyG-WHtR is significantly and linearly associated with CVD (30). Evidence regarding the association of baseline and modified TyG indices with incident CVD remains limited in CKM populations. A recent longitudinal study of individuals with CKM stages 0–3 demonstrated a linear relationship between triglyceride glucose-body mass index (TyG-BMI) and CVD risk (17). Furthermore, research utilizing the CHARLS database indicated correlations of cumulative TyG-WHtR with CVD outcomes; however, the cross-sectional design precludes causal inference (21). This study addressed this knowledge gap by incorporating four waves of longitudinal follow-up data and characterizing the dose-response relationship using restricted cubic splines. Notably, TyG-WHtR demonstrated a nonlinear relationship with CVD and identified a potential inflection point at 3.57 with CKM stages 0–3. To assess the robustness of our findings, we conducted sensitivity analyses by expanding the study population to include participants across all CKM stages 0–4. Furthermore, to reduce potential bias from short-term follow-up data, we performed additional analyses excluding participants with follow-up duration < 2 years. Despite these methodological refinements, the association between TyG-WHtR and CVD risk persisted with statistical significance. When TyG-WHtR exceeded 3.57, the risk of CVD increased significantly. Furthermore, given that CVD can change dynamically, the relationship may be influenced by factors such as sex and age (31). To investigate this, we performed additional subgroup analyses; however, no significant factors were identified that impact this relationship.

TyG-WHtR represents a novel marker significantly relevant to CVD. TyG is a well-established surrogate marker for insulin resistance and reflects associated metabolic consequences (32). Insulin resistance disrupts glucose metabolism, increases oxidative stress, induces systemic inflammation, promotes mitochondrial dysfunction, and contributes to dyslipidemia, endothelial dysfunction, and increased volume load, all of which influences the development of CVD (33). Additionally, triglycerides are known to accumulate within arterial walls, attracting inflammatory cells and further accelerating the development of atherosclerosis—the pathological foundation of many CVDs (34, 35). Additionally, insulin resistance and metabolic dysregulation induce endothelial dysfunction, characterized by decreased nitric oxide (NO) synthesis and bioavailability. This leads to vasoconstriction, platelet aggregation, and prothrombotic states, ultimately contributing to increased CVD risk (36). Additionally, WHtR is a more widely recognized marker of central obesity. Central obesity is strongly linked to various CVD risk factors, such as hypertension, atherosclerosis, and metabolic syndrome (21). Additionally, abdominal fat, known for its metabolic activity, secretes a variety of cytokines and inflammatory mediators, including adipokines. These substances contribute to chronic low-grade inflammation, endothelial dysfunction, and elevated blood pressure, all of which are critical in the development of CVD (37). The biological mechanisms linking TyG-WHtR to CVD risk encompass insulin resistance, metabolic dysregulation, inflammatory pathways, vascular dysfunction, and prothrombotic processes. Therefore, TyG-WHtR combines markers of insulin resistance and central obesity, thereby capturing the synergistic effects of these two interrelated mechanisms on CVD risk (38).

Compared with traditional methods for assessing insulin resistance (IR), the TyG index is more convenient, cost-effective, and easier to promote and apply in clinical practice (39). By monitoring the TyG index, high-risk individuals can be identified, enabling proactive preventive measures such as lifestyle interventions and pharmacological treatments (40). Regarding the practical application of TyG-WHtR in clinical practice and public health interventions, current research remains exploratory, and no unified clinical guidelines or large-scale intervention strategies have been established.

## 5 Strength and limitations of this study

This study includes a large sample size and a long-term follow-up cohort, with a nationally representative study population, ensuring its credibility. Notably, this is the first study to explore the longitudinal relationship between TyG-WHtR and CVD among middle-aged and older Chinese adults. However, certain methodological limitations warrant consideration: (1) CVD diagnoses were largely self-reported, which may introduce information bias due to misclassification. Although we supplemented with available treatment/medication data to improve specificity, some misclassification likely remains. This could bias effect estimates toward or away from the null and limit causal interpretation. Future work using clinically confirmed endpoints (medical records, registries, adjudicated outcomes) would help validate our findings. (2) Our sample comprises Chinese adults aged ≥45, limiting the generalizability of findings to younger individuals, other ethnicities, or different settings. The TyG-WHtR–CVD association may vary by age, genetics, culture, and healthcare context. We present our conclusions within the studied population and encourage replication in diverse, multiethnic cohorts. (3) Despite extensive adjustment, residual confounding from unmeasured factors (detailed dietary information, sedentary/activity patterns, family history, environmental exposures, psychosocial factors, broader medication use) may remain. Measurement error in covariates could further bias estimates. Future studies with richer lifestyle/genetic data and causal inference methods (IV, refined propensity scores, negative controls) are warranted to bolster causal interpretation.

## 6 Conclusion

This study confirms a positive association between TyG-WHtR and CVD in middle-aged and older Chinese adults with CKM stages 0–3. Evaluating the comparative predictive performance of TyG-WHtR vs. established and novel biomarkers across diverse age groups and racial/ethnic populations constitutes a critical area for future research.

## Data Availability

The datasets presented in this study can be found in online repositories. The names of the repository/repositories and accession number(s) can be found below: The datasets analysed during the this study are available in the China Health and Retirement Longitudinal Study repository (http://charls.pku.edu.cn).
