# Supplementary material for: The nonlinear association between the triglyceride glucose-waist-to-height ratio index and cardiovascular disease among middle-aged and older adults in China with cardiovascular-kidney-metabolic syndrome stages 0–3
Source: Front Cardiovasc Med. 2025 Aug 29;12:1604270. doi: 10.3389/fcvm.2025.1604270 (PMC12426055; doi:10.3389/fcvm.2025.1604270)
Supplement: Supplementary file 1 [file Table1.doc]

Table S1: Collinearity screening between TyG-WHtR and other variables.

|  | Variance inflation factor | | |
| --- | --- | --- | --- |
|  | Step 1 |  |  |
| TyG-WHtR | 1.7 |  |  |
| Sex | 3.2 |  |  |
| Married | 1.1 |  |  |
| Rural | 1.1 |  |  |
| Hypertension | 2.2 |  |  |
| Diabetes | 1.5 |  |  |
| Dyslipidemia | 1.1 |  |  |
| Drinking | 1.5 |  |  |
| Smoking | 2.2 |  |  |
| DBP | 3.4 |  |  |
| SBP | 2.6 |  |  |
| TC | 4.1 |  |  |
| HDLC | 1.5 |  |  |
| LDLC | 3.6 |  |  |
| CRP | 1 |  |  |
| Age | 1.7 |  |  |
| Education | 1.3 |  |  |
| HbA1c | 1.4 |  |  |
| BUN | 1.1 |  |  |
| UA | 1.4 |  |  |
| Medication history | 1.2 |  |  |
| CKM | 2.4 |  |  |

Note-1: Variance inflation factor = 1/(1-R^2^). Abbreviations as in Table 1.

Note-2: The variables with variance inflation factor >5 will be regarded as collinear variables and cannot be included in the multiple regression model.

**Table S2 Multivariate Cox regression analyses for the association between TyG-WHtR and CVD across CKM stages 0-4.**

|  |  | |  | **HR (95%CI)** | |  |  | |
| --- | --- | --- | --- | --- | --- | --- | --- | --- |
|  | **Model 1** |  |  | **Model 2** |  |  | **Model 3** |  |
| TyG-WHtR | 1.29 (1.22, 1.37) |  |  | 1.11 (1.04, 1.18) |  |  | 1.10 (1.02, 1.19) |  |
| Q1 | Ref |  |  | Ref |  |  | Ref |  |
| Q2 | 1.19 (1.02, 1.38) |  |  | 1.14 (0.98, 1.33) |  |  | 1.13 (0.96, 1.32) |  |
| Q3 | 1.37 (1.18, 1.59) |  |  | 1.18 (1.02, 1.38) |  |  | 1.16 (0.99, 1.36) |  |
| Q4 | 1.80 (1.56, 2.08) |  |  | 1.33 (1.14, 1.56) |  |  | 1.30 (1.09, 1.56) |  |
| *P*-trend | <0.001 |  |  | <0.001 |  |  | <0.001 |  |

Model 1: No covariates were adjusted. Model 2: Age, sex, marital status, rural, hypertension, dyslipidemia, diabetes, drinking status, smoking status, CKM, medication history, SBP, DBP were adjusted. Model 3: HDL-C, LDL-C, TC, UA, BUN, HbA1c and CRP were further adjusted based on model 2.

**Table S3 Multivariate Cox regression analyses for the association between TyG-WHtR and CVD Among Individuals With Follow-Up >2 Years**

|  |  | |  | **HR (95%CI)** | |  |  | |
| --- | --- | --- | --- | --- | --- | --- | --- | --- |
|  | **Model 1** |  |  | **Model 2** |  |  | **Model 3** |  |
| TyG-WHtR | 1.29 (1.22, 1.37) <0.0001 |  |  | 1.11 (1.04, 1.18) 0.0021 |  |  | 1.09 (1.01, 1.18) 0.0298 |  |
| Q1 | Ref |  |  | Ref |  |  | Ref |  |
| Q2 | 1.19 (1.02, 1.38) |  |  | 1.14 (0.98, 1.33) |  |  | 1.12 (0.96, 1.31) |  |
| Q3 | 1.37 (1.18, 1.58) |  |  | 1.18 (1.02, 1.38) |  |  | 1.15 (0.98, 1.35) |  |
| Q4 | 1.80 (1.56, 2.07) |  |  | 1.33 (1.14, 1.56) |  |  | 1.28 (1.07, 1.54) |  |
| *P*-trend | <0.001 |  |  | <0.001 |  |  | <0.001 |  |

Model 1: None covariates were adjusted. Model 2: Age, sex, marital status, rural, hypertension, dyslipidemia, diabetes, drinking status, smoking status, CKM, drug history, SBP, DBP were adjusted. Model 3: HDL-C, LDL-C, TC, UA, BUN, HbA1c and CRP were further adjusted based on model 2.
